# Engineering Spiking Neurons Using Threshold Switching Devices for High-Efficient Neuromorphic Computing

**DOI:** 10.3389/fnins.2021.786694

**Published:** 2022-01-05

**Authors:** Yanting Ding, Yajun Zhang, Xumeng Zhang, Pei Chen, Zefeng Zhang, Yue Yang, Lingli Cheng, Chen Mu, Ming Wang, Du Xiang, Guangjian Wu, Keji Zhou, Zhe Yuan, Qi Liu

**Affiliations:** ^1^Frontier Institute of Chip and System, Fudan University, Shanghai, China; ^2^State Key Laboratory of ASIC and System, School of Microelectronics, Fudan University, Shanghai, China; ^3^Department of Physics, Center for Advanced Quantum Studies, Beijing Normal University, Beijing, China; ^4^Shanghai Qi Zhi Institute, Shanghai, China; ^5^Key Laboratory of Microelectronic Devices and Integrated Technology, Institute of Microelectronics, Chinese Academy of Sciences, Beijing, China

**Keywords:** threshold switching devices, influence factors, spiking neuron circuits, frequency tunability, spiking neural network, reinforcement learning

## Abstract

Inspired by the human brain, the spike-based neuromorphic system has attracted strong research enthusiasm because of the high energy efficiency and powerful computational capability, in which the spiking neurons and plastic synapses are two fundamental building blocks. Recently, two-terminal threshold switching (TS) devices have been regarded as promising candidates for building spiking neurons in hardware. However, how circuit parameters affect the spiking behavior of TS-based neurons is still an open question. Here, based on a leaky integrate-and-fire (LIF) neuron circuit, we systematically study the effect of both the extrinsic and intrinsic factors of NbO_*x*_ -based TS neurons on their spiking behaviors. The extrinsic influence factors contain input intensities, connected synaptic weights, and parallel capacitances. To illustrate the effect of intrinsic factors, including the threshold voltage, holding voltage, and high/low resistance states of NbO_*x*_ devices, we propose an empirical model of the fabricated NbO_*x*_ devices, fitting well with the experimental results. The results indicate that with enhancing the input intensity, the spiking frequency increases first then decreases after reaching a peak value. Except for the connected synaptic weights, all other parameters can modulate the spiking peak frequency under high enough input intensity. Also, the relationship between energy consumption per spike and frequency of the neuron cell is further studied, leading guidance to design neuron circuits in a system to obtain the lowest energy consumption. At last, to demonstrate the practical applications of TS-based neurons, we construct a spiking neural network (SNN) to control the cart-pole using reinforcement learning, obtaining a reward score up to 450. This work provides valuable guidance on building compact LIF neurons based on TS devices and further bolsters the construction of high-efficiency neuromorphic systems.

## Introduction

In the big data era, traditional computing architectures are facing the challenge known as the “Von Neumann bottleneck” due to the separated memory and computing units and thus struggling on high efficiency to process massive data (Ambrogio et al., 2018; Zidan et al., 2018; Sebastian et al., 2020). By contrast, neuromorphic systems based on the spiking neural network (SNN) integrate collocated memory and computing components, eliminating the memory bottleneck and data congestion (Wang et al., 2014; Kheradpisheh and Masquelier, 2020; Taherkhani et al., 2020). Also, combining the event-driven and spatiotemporal information processing features, such a system shows great potential to build a low-power and high-efficiency machine for handling different types of data-intensive tasks, thus attracting much attention in both the industry and academia field.

For implementing the SNN hardware system, two key modules must be developed: artificial synapses and spiking neurons. Artificial synapses connect pre- and post-neurons and store weight values that determine the conveyed information intensity (Werner et al., 2016; Zhang et al., 2017; Prezioso et al., 2018; Kurenkov et al., 2019). Recently, emerging memories such as phase-change memory (PCM) (Nomura et al., 2019; Nandakumar et al., 2020), resistive random-access memory (RRAM) (Yu et al., 2015; Guo et al., 2019; Duan et al., 2020; Kim et al., 2021; Shi et al., 2021), and magnetoresistance memory (Zhang et al., 2021a) in a crossbar array structure are regarded as promising candidates to serve as artificial synapses. Neurons integrate and process the signals from the synaptic array and then transmit the resulting pulse to the next neuron. Neuron circuits based on complementary metal-oxide-semiconductor (CMOS) technology have been widely reported (Wijekoon and Dudek, 2008; Ebong and Mazumder, 2012; Cai et al., 2019). However, due to the limited biodynamics in CMOS devices, the constructed neuron circuit typically needs a large number of transistors and capacitors, accounting for a complex configuration, large area fraction in the overall chip design, and low integration density (Indiveri et al., 2006; Dutta et al., 2017; Prezioso et al., 2018). To improve the area efficiency and integration density of the neuromorphic hardware, emerging devices, such as PCM (Burr et al., 2010; Tuma et al., 2016) and RRAM (Pickett et al., 2013; Zhang et al., 2018, 2021b; Wang et al., 2021), have also been proposed to build spiking neurons. According to the retention time, the emerging neuron devices could be roughly divided into nonvolatile and volatile types. PCM is a typical nonvolatile memory with a metal-sulfide-metal structure, whose resistance change under pulse stimuli emulates the neuron’s membrane potential. However, due to the nonvolatile mechanism, PCM-based neurons inevitably lack leaky neural dynamics and demand additional reset circuits that introduce hardware overhead. In comparison, threshold switching (TS) devices perform abrupt resistance switching in a volatile manner. They switch to a low-resistance state (LRS) when the applied bias exceeds a threshold voltage (*V*_*th*_) and subsequently return to a high-resistance state (HRS) as the voltage drops below a hold voltage (*V*_*hold*_). Generally, TS-based neurons combine a simple TS device with a capacitor or resistor, which are equipped with the characteristic of self-sustained oscillation (Gao et al., 2017; Woo et al., 2019; Wang et al., 2020). Such a neuron circuit allows the design of an inductor-free circuit without needing an additional reset circuit, which has the advantages of low power consumption, nanoscale scalability, and high integration intensity. To promote TS-based neurons in practical applications, deep research in the influence parameters of their firing responses is urgent. It has been reported that external circuit elements such as capacitance (*C*) and resistance can affect the firing rates in some certainty (Ignatov et al., 2015; Duan et al., 2020; Zhang et al., 2020). However, the relationship between the intrinsic parameters (such as *V*_*th*_, *V*_*hold*_, LRS, and HRS) of the device and spike behaviors as well as the energy consumption is rarely studied. Thus, construction of a complete performance assessment system for TS-based neurons deserves more attention.

In this work, combining experiment and simulation methods, we comprehensively investigate the spiking frequency characteristic of leaky integrate-and-fire (LIF) neurons based on a NbO_*x*_-based TS device. First, the effect of extrinsic circuit parameters, such as the synaptic weights that connect with the neuron, and the membrane *C*, on the spiking frequency are studied by controlling the input intensity. Second, with the help of the proposed device model, we further illustrate how the intrinsic parameters (*V*_*th*_, *V*_*hold*_, *R*_*high*_, and *R*_*low*_) affect the spiking behaviors. These results offer a thorough understanding of the intrinsic factors of neurons that determine the firing frequency, further providing device engineering methods for controlling the firing response of neurons. For further building low-power neuromorphic computing systems using these neuron circuits, the relationship between firing rate and the energy consumption per spike is also discussed. Finally, we construct an SNN with the structure of 8 × 256 × 2 for cart-pole control and achieves up to 450 reward score, demonstrating the practical application of TS-based neurons. This work shows comprehensive guidance on building spiking neurons with TS devices and has great significance in developing high-efficient neuromorphic systems using emerging devices.

## Materials and Methods

### Device Fabrication

The fabrication processes of the Ti/Pt/NbO_*x*_/Ti/Pt device are as follows. First, the bottom electrode composed of Pt film with a thickness of 40 nm and Ti adhesive layer with a thickness of 5 nm was deposited by electron beam evaporation. The patterning was processed by photolithography and released by liftoff processes. Then, the pattern of the functional layer was formed by the second lithography process. And then, NbO_*x*_ (2 < *x* < 2.5) with a thickness of 50 nm was deposited by magnetron sputtering. Next, the liftoff process was carried out. Finally, Ti film with a thickness of 10 nm was deposited as the top electrode and capped by a 30-nm Pt protection layer by magnetron sputtering, and then the device was released by the final liftoff process. The area of the devices is 5 μm × 5 μm.

### Electrical Measurements

During the test of the device’s electrical characteristics, a Keithley 4200 semiconductor parameter analyzer was used to provide input signals and detect the current flowing through the NbO_*x*_ device. The voltage bias was applied to the top electrodes of the NbO_*x*_ device, and the bottom electrodes were grounded. During testing of the spiking behavior of the neuron circuit, a Keithley 4200 supplied the input voltage pulse and measured the current flowing through the NbO_*x*_ device. A Tektronix oscilloscope was used to measure the voltage oscillation output on the NbO_*x*_ device.

### Simulations

In this research, we construct an empirical device model of the NbO_*x*_ TS device based on the experimental data. The NbO_*x*_ TS device is a two-terminal device with two resistive states—HRS and LRS, whose state transition depends on the applied voltage. We used a piecewise function to fit the experimental results of the resistance of the device varying with the input voltage. The detailed process is described in the following functions (Eqs. 1 and 2):


(1) $${\text{R}}_{\mathrm{high}}={\text{R}}_{0}-\text{a}\,\text{exp}\,\frac{\text{v}+\text{b}}{\text{c}}$$

(2) $${\text{R}}_{\mathrm{low}}={\text{R}}_{1}+\text{m}\,\text{exp}\,\left(-\text{nv}\right)$$

where *R*_*high*_ and *R*_*low*_ are the HRS and LRS, respectively, *v* is the input voltage, and a, b, c, m, and n are fitting parameters. Initially, the device resistance-voltage function follows Eq. 1. When the voltage applied to the device exceeds *V*_*th*_, the device switches from the HRS to the LRS and the resistance-voltage relation obeys Eq. 2. Then, as the input stimulus decreases below *V*_*hold*_, the device switches back from an LRS to an HRS again, and the resistance varies with the voltage according to Eq. 1 (see Supplementary Figure 1 for the R-V fitting curve). In addition, biological neurons are stochastic in nature; the stochastics in TS-based neuron is mainly responsible for the threshold fluctuation. Hence, we extract the distribution of *V*_*th*_ and *V*_*hold*_ of the device from the measured data, proving to be Gaussian distribution (see Supplementary Figure 2). Thus, we use a Gaussian distribution rather than a fixed value for *V*_*th*_ and *V*_*hold*_ in the simulation.

The simulated LIF neuron circuit was made of a resistor and a NbO_*x*_ memristor in parallel with a capacitor. According to the law of Kirchhoff, the relation between the voltage across the device and the input can be described as (Eq. 3):


(3) $$\text{C}\,\frac{{\text{dV}}_{{\mathrm{NbO}}_{x}}\,\left(t\right)}{\text{dt}}=\frac{\text{V}-{\text{V}}_{{\mathrm{NbO}}_{x}}\,\left(t\right)}{\text{R}}-\frac{{\text{V}}_{{\mathrm{NbO}}_{x}}\,\left(t\right)}{{\text{R}}_{{\mathrm{NbO}}_{x}}}$$

Thus, the simulated spiking behavior is obtained based on Eq. 3.

## Results

### Schematic of a Biological Neuron and a NbO_*x*_-Based Leaky Integrate-and-Fire Neuron

**FIGURE 1:**
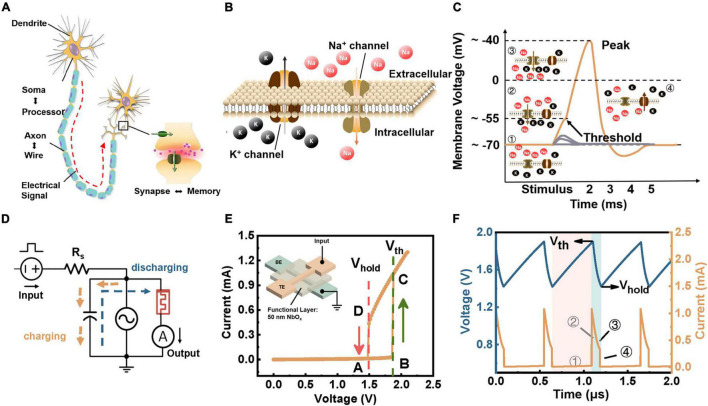
Biological neuron vs. artificial neuron. **(A)** Schematic of biological neurons, which can be divided into three parts: dendritic, soma, and axon. **(B)** Illustration of a cell membrane embedded with ion channels letting charged particles enter or leave a cell to produce electrical signals. **(C)** Diagram of an action potential generation. **(D)** Circuit configuration of a TS-based LIF neuron circuit, consisting of a synaptic resistor (*R_s*) and a TS device with a parallel capacitance (*C*) that is either parasitic or external. **(E)** Hysteresis I-V characteristic of a NbO_*x*_-based TS device, which is composed of a NbO_*x*_ functional film deposited onto a Ti/Pt layer as bottom electrode to form a sandwich that is covered with another Ti/Pt layer as top electrode. **(F)** Voltage output waveform and current spike of the TS-based LIF neuron circuit under a constant bias.

Figure 1A shows the schematic of a simple connected biological neural network consisting of neurons and connected synapses. Concisely, a single neuron contains three functional parts: dendrites that receive pre-neurons’ inputs, soma that serves as the central processing part, and the axon that generates and transmits action potentials. Figure 1B illustrates the schematic diagram of the cell membrane. Once the cell receives stimuli, ion channels enable the ions (such as Na^+^ and K^+^) to flow through the cell membrane, further leading to potential differences on both sides of the membrane. Figure 1C demonstrates the concrete production process of an action potential. At the initial stage (➀ in Figure 1C), the neuron remains in a resting state. When the neuron receives an external stimulation, several Na^+^ channels open so that a small amount of Na^+^ flows into the membrane and lifts the membrane potential (➁ in Figure 1C). During stimulus intervals, the membrane potential gradually falls back to a polarized state spontaneously due to the continual ion exchange through the membrane, corresponding to leaky features (gray dotted line in Figure 1C). As more stimulation is applied to the neuron, more Na^+^ channels open, enabling more Na^+^ to enter the cell and further increasing membrane potential and generating impulses when it exceeds the threshold (➂ in Figure 1C). After that, Na^+^ channels are closed while K^+^ channels are opened up to cause the outflow of K^+^, inducing the depolarization (➃ in Figure 1C). That is the whole generation process of an action potential (Kandel et al., 2000).

The LIF model is a simplified neuron model that follows the working principle of biological neurons in some certainty and provides a feasible solution for spike generation (Burkitt, 2006). In the LIF circuit model, the cell membrane is similar to a capacitor, which integrates charges injected into the neuron. A resistor parallel with the capacitor performs the leaky behavior. A switch driven by input is supposed to take the place of ion channels. In such a model, the resistor and switch could be faithfully implemented in a TS device (such as the NbO_*x*_ device). Therefore, combining the dynamics of a TS device with a capacitor could emulate the LIF neurons in an abstract form, as shown in Figure 1D. The circuit comprises a synaptic resistor (*R_s*) and a TS device in parallel with a *C* that can be either the external or the intrinsic *C*. There have been many studies for building such a spiking neuron with self-sustained spikes or oscillations based on TS devices.

Here, we study the LIF neuron using a typical NbO_*x*_-based TS device, where the threshold fluctuations in the dynamics are utilized as inherent physical noise to implement the stochasticity. The schematic of the device with Ti/Pt/NbO_*x*_/Ti/Pt structure is illustrated in the inset of Figure 1E. Figure 1E depicts the current-voltage (I-V) characteristics of the device (see Supplementary Figure 3 for current scan curve). Initially, the device is in an HRS, which switches to an LRS (B→C) once the voltage reaches a threshold value V_*th*_. When the voltage is lower than the hold voltage (V_*hold*_), the device spontaneously returns to the HRS (D→A) (see Supplementary Figure 1 for R-V characteristic). Thus, the device is capable of emulating the open–close dynamics of ion channels in biological neurons. We also demonstrate that such a TS device exhibits more than 10^12^ cycles and thus possesses the potential to construct artificial neurons (Zhang et al., 2020). Figure 1F exhibits the spiking behavior of the neuron circuit based on such a device under a constant voltage input. During working, the capacitor charges through the connected R_*s*_ and lifts up the voltages on the NbO_*x*_ devices. When the voltage on the device cannot exceed the *V*_*th*_ of ∼1.90 V, the device’s current varies from A to B as the voltage increases. The device remains in HRS and I_NbOx_ is subtle (➀ in Figure 1F). Once the bias further rises up to *V*_*th*_, the device switches to an LRS abruptly, and the current flowing through the device experiences a significant increase from B to C in Figure 1E (➁ in Figure 1F). Then, the capacitor discharges through the NbO_*x*_ device, and the current decays along the C→D curve in Figure 1E (➂ in Figure 1F), where the device remains in LRS. Once the voltage drops below the holding voltage (*V*_*hold*_) of ∼1.42 V, the device returns to HRS spontaneously so that the current flowing through the device falls off from D to A in Figure 1E (➃ in Figure 1F). Therefore, the neuron generates a complete current spike and prepares for the next spike event.

### The Spiking Response of the Firing Neuron Under Different Parameters

In biological nervous systems, the firing frequency of neuron pulse is often used to encode information (Adrian and Zotterman, 1926; Chapleau, 2007). Hence, it is attractive to explore the spiking response of a neuron circuit and further control it by changing the input or other related circuit parameters. Then, we study the intensity-modulation spiking characteristics based on the abovementioned neuron circuit. For a clearer presentation, we split the analysis of the influencing factors into two parts. First, we discuss the effect of external parameters on the firing frequency, i.e., the input strength, *R*_*s*_, and *C* in the circuit. After that, we explore the influence of the device’s intrinsic parameters, including *V*_*th*_, *V*_*hold*_, HRS, and LRS.

#### Effect of External Circuit Parameters on Spiking Behaviors

**FIGURE 2:**
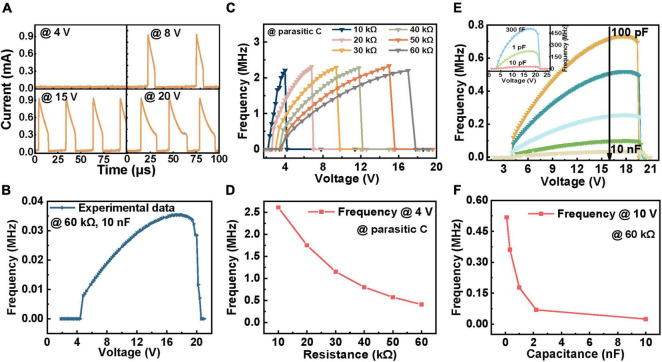
The influence of firing rates via changing input intensity (*V*_*in*_), synaptic resistor (*R*_*s*_) and capacitance (*C*). The device used has a *V*_*th*_ of ∼1.90 V and a *V*_*hold*_ of ∼1.42 V. **(A)** The firing response under different *V*_*in*_ (4, 8, 15, and 20 V). The circuit parameters are as follows: *R_*s*_* = 60 kΩ, *C* = 10 nF. **(B)** Corresponding frequency-voltage curve. **(C)** The relation between frequency and voltage as a function of *R*_*s*_ under parasitic capacitance. **(D)** The oscillation behavior with different *R*_*s*_ under the same *V*_*in*_ of 4 V. **(E)** The relation between frequency and voltage as a function of *C* under *R*_*s*_ = 60 kΩ, which was reduced to 330 fF in simulation and the firing response frequency of which is demonstrated in the inset. **(F)** The oscillation behavior with different *C* under the same *V*_*in*_ of 10 V.

Figure 2A illustrates the spike output of a fixed neuron circuit under different *V*_*in*_. The input signals are single voltage pulses with amplitudes varying from 2 to 21 V. When *V*_*in*_ is less than 4 V, the V_NbOx_ is insufficient to switch the device on and thus presents no spike output during the time interval studied. When *V*_*in*_ = 8 V, 15 V, 20 V, V_NbOx_ exceeds *V*_*th*_ and switches the device on. To obviously present the relation between the firing rate and *V*_*in*_, we plot the frequency as a function of input intensity, as shown in Figure 2B. The spiking frequency amplifies first and then attenuates with the strengthening of *V*_*in*_, which faithfully realizes the response of biological neurons (Stetler et al., 2009). The appearance of the saturated frequency can be attributed to the counteraction of the decrease of integration time and the increase of discharge time with increasing the input intensity. Under low input intensity, the integration time dominates the output frequency until the integration time equals the discharge time, where the frequency gets saturated (see Supplementary Figure 4 for detailed variation of both the integration time and discharge time).

Previous studies have roughly indicated that the spiking frequency is affected by *R*_*s*_ and *C* (Liu et al., 2016). To formally and systematically verify this conclusion, a set of voltage pulses ranging from 0 to 25 V was applied to the same neuron under a single variable of *R*_*s*_ or *C*. Figure 2C shows the relationship between the firing frequency and different *V*_*in*_ with varying the *R*_*s*_. Under the same *V*_*in*_, the frequency decreases with increasing *R*_*s*_ (Figure 2D). This phenomenon can be clarified as that a larger *R*_*s*_ resulting in a higher time constant and thus extends the integration time that directly affects the firing rate of the device (Zhang et al., 2020). However, the essence of the slow-down effect is the decrease of charging current caused by the increase of *R*_*s*_, which can be offset by lifting the input intensity. Thus, the saturation values of output frequency are identical (see Supplementary Figure 5 for detailed integration and discharge time with different *R*_*s*_). Figure 2E demonstrates the firing frequency of circuits with different *C* under different *V*_*in*_. For each curve, the frequency sustains the increase until it reaches the peak value with intensifying *V*_*in*_. At the same *V*_*in*_, the spiking frequency decreases with the growth of *C*, as shown in Figure 2F. This is because the increase of *C* causes the expansion of both integration time constant and discharge time constant, which also results in the reduction of peak frequency under a larger *C*. This cannot be eliminated by varying *V*_*in*_ (see Supplementary Figure 6 for a more detailed analysis).

Furthermore, to explore the limiting frequency of the device, we shrink *C* to 300 fF in our circuit model in view of the inevitable wire parasitic *C* in the actual test. The inset of Figure 2E illustrates the spike frequency under different *V*_*in*_ when *C* declines to 10 pF, 1 pF, and 300 fF. The results reveal that the spiking frequency can be as high as 500 MHz under 300 fF *C*. Such a high oscillation frequency is expected to promote the rapid development of computing using neuron-like spike signals. As *C* is minimized to several orders of fF, the charging/discharge time of the device is as small as the transition time; then, the peak frequency is restricted by the transition time of the device. Also, during the period of transition from HRS (LRS) to LRS (HRS) of the device, an overshoot (undershoot) above *V*_*th*_ (below *V*_*hold*_) of the output oscillation voltage is consequently generated (see Supplementary Figure 7 for the spike train schematic of the device with transition time) (Chen et al., 2016). The results show that the spiking frequency of neurons can be regulated *via* changing stimuli, synaptic weights, and membrane *C* to modify the integration and discharge time. Specifically, the *C* determined the peak frequency, while the synaptic weights did not. This enables us to customize the properties by adjusting circuit parameters to meet the demands of specific applications.

#### Effect of Intrinsic Parameters on Spiking Behaviors

**FIGURE 3:**
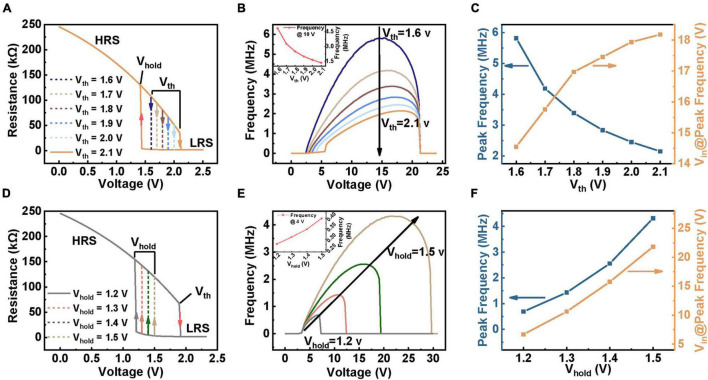
The simulation results of the influence of spiking behavior through varying *V*_*th*_ **(A–C)** and *V*_*hold*_ **(D–F)**. The circuit parameters used in the simulation are: *C* = 100 pF, *R*_*s*_ = 60 kΩ. **(A)** Schematic diagram of changing *V*_*th*_ in simulation with a ∼1.42 V *V*_*hold*_. **(B)** Related firing frequency with different *V*_*th*_. **(C)** The plot of peak frequency and corresponding voltage with *V*_*th*_. **(D)** Schematic diagram of changing *V*_*hold*_ in simulation with a ∼1.90 V *V*_*th*_. **(E)** Related frequency with different *V*_*hold*_. **(F)** The effect of *V*_*hold*_ on peak frequency and required voltage.

**FIGURE 4:**
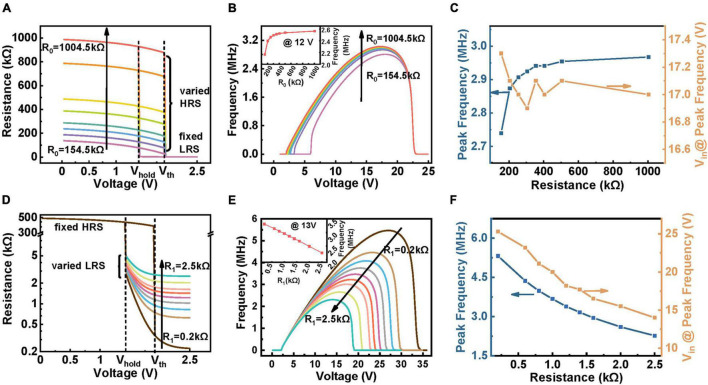
Illustration of the variation of firing response caused by *R*_*high*_ **(A–C)** and *R*_*low*_ **(D–F)** in the developed model. The circuit parameters used in the simulation are: *C* = 100 pF, *R*_*s*_ = 60 kΩ **(A)** Different values of HRS adjusted in the model. The linear part of *R*_*high*_ function, *R*_0_, are marked on the figure, from bottom to top: 154.5, 204.5, 254.5, 304.5, 404.5, 504.5, 804.5, 1004.5 kΩ. Changing *R*_0_ is actually equivalent to shifting the R-V curve in the HRS along the *Y*-axis. **(B,C)** The simulation results of difference between frequency, peak firing behavior and required V_*in*_ brought about by changing *R*_*high*_. **(D)** The tuning of *R*_*low*_ in the model. *R*_1_, which is the linear part of *R*_*low*_ function is noted above, along the direction of the arrow is: 0.2, 0.6, 0.8, 1, 1.2, 1.4, 1.6, 2, 2.5 kΩ. **(E,F)** Related results obtained in **(D)**.

A vital aim of this research is to guide device screening or design according to the frequency required for application. Therefore, it is critical to figure out the relationship between device parameters and spiking frequency. In this part, four intrinsic factors that affect the spiking frequency are studied based on our model: (I) *V*_*th*_ and *V*_*hold*_ of the device (Figure 3); (II) HRS and LRS of the device (Figure 4).

(I) Effect of device’s *V*_*th*_ and *V*_*hold*_ on spiking behavior.

Figure 3A shows a schematic of the *V*_*th*_ ranging from 1.6 to 2.1 V with a fixed V_*hold*_. The recorded frequency-voltage curves corresponding to each given *V*_*th*_ are illustrated in Figure 3B. The results illustrate that the beginning spiking voltage (BSV) increases with increasing *V*_*th*_, but the stop spiking voltages (SSVs) are unchanged. This is directly attributed to the requirement that the device with higher *V*_*th*_ needs to be switched on at a higher *V*_*in*_. The inset of Figure 3B presents that the spiking frequency decreases with increasing *V*_*th*_ under the same *V*_*in*_. This mainly results from the prolonged time for the integration process and also the increased time for discharging. Besides, the longer integration and discharge time caused by the increased *V*_*th*_ cannot be fully compensated by a higher *V*_*in*_, resulting in a difference in peak firing frequency, as revealed in the blue curve in Figure 3C. On the other hand, the orange curve in Figure 3C illustrates that the voltage demanded by the saturation frequency gradually increases with a higher *V*_*th*_. This is because the extension of integration time calls for a higher bias to compensate for the variation of integration and discharge time (see Supplementary Figure 8 for detailed discussion).

Except for the *V*_*th*_, we further adjust the device with different *V*_*hold*_, as shown in Figure 3D. Figure 3E shows the corresponding frequency response curves under different *V*_*in*_. Unlike changing *V*_*th*_, which affects the BSV, the change of *V*_*hold*_ brings about different SSVs. This is attributed to that a lower *V*_*hold*_ requires the device to be turned off at a lower *V*_*in*_, leading to the decline of stop voltages. The spiking frequency of the device with different *V*_*hold*_ under the same input is shown in the inset of Figure 3E. Obviously, the increase of *V*_*hold*_ generates a higher frequency, which could be explained by the contraction of both charging and discharge time, thus shrinking the oscillation window. To more clearly present the effect of V_*hold*_ on spiking behaviors, we extracted the peak frequency under different input intensities. In contrast to the effect of *V*_*th*_, the peak frequency increases with enhancing the *V*_*hold*_, as shown in Figure 3F and Supplementary Figure 9. A higher input voltage is also required to obtain the peak frequency with the increase of *V*_*hold*_. These results demonstrate that to obtain a higher peak frequency, designing a device with narrow *V*_*th*_-*V*_*hold*_ window and low operation voltages is required.

(II) Effect of device’s HRS and LRS on spiking behavior.

During working, the TS device in the neuron circuit needs to switch frequently between HRS and LRS to complete the continuous spiking process; hence, these two states are important factors. Both high (*R*_*high*_) and low (*R*_*low*_) resistances are functions of voltage (see section “Materials and Methods” for details). For providing qualitative analysis, only the constant part value of HRS (*R*_0_) and LRS (*R*_1_) is changed here, which corresponds to HRS (LRS) shifts along the ordinate, as shown in Figures 4A,D, respectively. Figure 4B demonstrates the relation of frequency and *V*_*in*_ as a function of *R*_0_ obtained through simulation. Obviously, the larger *R*_0_ enables V_NbOx_ to reach *V*_*th*_ at a lower *V*_*in*_ due to the partial-voltage effect, so the BSV tends to shift to the left while the SSV remains unchanged. In addition, the frequency increases with increasing *R*_0_ under the same *V*_*in*_ (inset in Figure 4B), which can be explained by the effect of *R*_*high*_ on the leaky of *C* during charging, i.e., higher *R*_*high*_ leads to less leaky and thus shortens the integration process. Moreover, to directly present the effect of *R*_0_ on the peak spiking behavior, we also plot the peak frequency-*R*_0_ and V_*in*_-*R*_0_ relationship curves in Figure 4C. As *R*_0_ enhances, the peak frequency first increases slightly and then tends to be saturated (orange curve in Figure 4C, see Supplementary Figure 10 for detailed explanation), while the *V_*in*_@peak frequency* is nearly identical. The results show that the HRS (*R*_0_) only has a slight effect on the peak frequency, which may help to fine modulate the peak frequency of the circuit.

Likewise, as for the case of changing *R*_1_, the recorded frequency-*V*_*in*_ relation is illustrated in Figure 4E. Apparently, the *R*_1_ presents a more dominant effect on spiking frequency than *R*_0_. With increasing the *R*_1_, both the SSV and peak frequency decrease while the BSV remains unchanged. This is because the discharging time is longer under the same input when the R1 is higher, which reduces spiking frequency. Also, a higher *R*_1_ divides higher voltage on the device, making the circuit stop firing under a lower input voltage. The inset of Figure 4E shows the frequency evolution with decreasing the *R*_1_, indicating a linear relationship. Correspondingly, the peak frequency-*R*_1_ and V_*in*_-*R*_1_ relationship curves are illustrated in Figure 4F. Both the peak frequency and *V_*in*_@peak frequency* decline as the *R*_*low*_ increases (see Supplementary Figure 11 for detailed explanation). These results suggest that the *R*_*low*_ is an effective factor in modulating the spiking frequency of the neuron circuit, providing us the guidance to design TS device with decent *R*_*low*_ to meet the practical applications.

From now on, various parameters of modulating the firing frequency of spiking neurons have been studied separately. To more intuitively present the evolution rule, we summarize the change of frequency, peak frequency, and *V_*in*_@peak frequency* as these parameters increase in Table 1.

**TABLE 1:** Summary of parameter influences on neuron spiking behaviors.

Circuit parameters			Frequency	Peak frequency	*V*_*in*_ @ Peak frequency
Extrinsic		*V*_*in*_	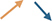	–	–
		*R*_*s*_	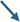	Unchanged	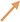
		*C*	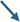	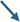	Unchanged
Intrinsic		*V*_*th*_	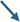	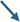	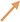
		*V*_*hold*_	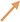	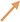	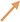
		*R*_*high*_	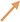	Slight increase followed by saturation	Slight decrease followed by saturation
		*R*_*low*_	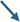	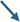	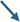

### The Influence Factors on Power Consumption Per Spike

Heat dissipation becomes the most serious challenge in the chip industry when it comes to the complex algorithms and architectures used in current machine learning tasks. Neuromorphic chips with bionic characteristics are regarded as a promising solution to reduce energy consumption (Blouw et al., 2019). In this context, for further expanding the practical applications of TS-based neurons, energy consumption is an inevitable evaluation parameter while optimizing the spiking frequency based on the application scenarios. Therefore, the following part presents the relationship between frequency and spike energy consumption so as to provide a reference for optimizing the firing states of neuron circuits.

**FIGURE 5:**
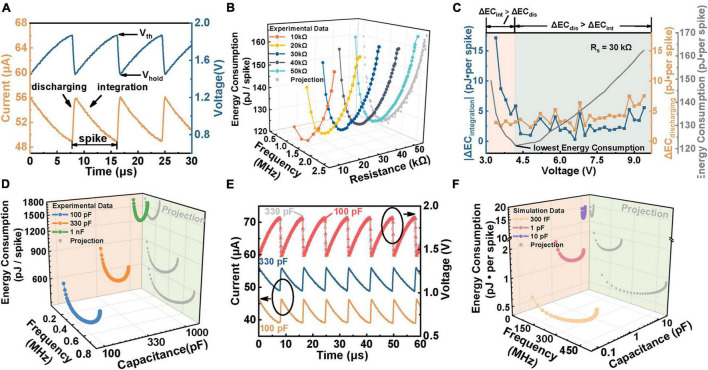
The energy consumption of the neuron cell. **(A)** Diagram of integration and discharge energy consumption calculation in a single spike. The orange curve represents the current flowing into the neuron cell node, and the blue curve represents the voltage applied to the neuron cell. The circuit parameters are: *C* = 330 pF, *R*_*s*_ = 60 kΩ, *V*_*in*_ = 5 V. **(B)** The variation of energy consumption per spike with spiking frequency related to *R*_*s*_ under a 100 pF *C*. **(C)** Analysis of the variation of integration time and discharge time, which are both extracted from **(B)**. Each data point in **(B)** is compared to the previous adjacent data point to obtain the changing value. EC is the abbreviation of energy consumption and “Δ” represents the changing size. Δ*EC_*int*_* and Δ*EC_*dis*_* refer to the variation of integration and discharge energy consumption, respectively. **(D)** The plot of energy consumption varied with frequency related to the parallel capacitance (*C*) ranged from 100 pF to 1 nF under a 60 kΩ *R*_*s*_, which are all experimental data. **(E)** Explanation of the increasing tendency of energy consumption with larger *C* in the inset of **(D)**. The orange and blue curves represent the overall current flowing through the neuron cell when the parallel *C* is 100 and 330 pF, respectively. The red and gray curves demonstrate the voltage across neuron cell when *C* is 100 and 330 pF, respectively. **(F)** Simulation plots of energy consumption-frequency when *C* is decreased to 330 fF.

According to the above analysis, capacitors play the role of the cell membrane, and the NbO_*x*_-based TS device emulates ion channels. In consequence, capacitor and TS devices are regarded as a whole, namely, neuron cells. Thus, to obtain the optimal energy efficiency of a system, we investigate the power consumption variations of a specific neuron cell brought about by varying *R*_*s*_ and *V*_*in*_. For calculating the total energy consumption of the neuron cell, the current flowing through *R*_*s*_ (which is equal to the current flowing through the cell according to Kirchhoff’s law) and the voltage across the device (which is equivalent to the voltage applied to the neuron cell) are measured. Figure 5A shows the measured total current and voltage on the neuron cell during oscillation. The process of V_NbOx_ from *V*_*hold*_ to *V*_*th*_ is defined as the capacitor integration process, the voltage on *R*_*s*_ hence decreases, resulting in the decline of current. The process of V_NbOx_ from *V*_*th*_ to *V*_*hold*_ is defined as the discharging process, and the voltage on *R*_*s*_ increases because the TS device switches on. Based on such data, we adopt the product of total current (the current flowing through the *R*_*s*_) and voltage on the neuron cell as the total power. Then we integrate the total power over an oscillation cycle to obtain the energy consumption of each spike under different conditions. Figure 5B shows the energy consumption of each spike under different *R*_*s*_ and fixed *C* (100 pF) with increasing the spike frequency (equal to increasing the *V*_*in*_). The energy consumption of neuron cell experiences an attenuation first and then an increase as the frequency increases. For each fixed *R*_*s*_, there is minimum energy consumption, and the minimum values are identical, which means that a determined neuron cell possesses a specified lowest energy consumption when the input intensity is decent. Moreover, the gray curve in Figure 5B is the projection of energy consumption per spike-frequency curve of multiple resistors in the ZX plane, indicating that energy consumption of the cell module is solely related to frequency. This phenomenon results from the offset between the variation of integration power consumption and discharge power consumption. In detail, as the frequency increases, the integration energy consumption decays continuously due to the shrinking of the integration process, while the discharging energy consumption gradually increases owing to the prolonged relaxation period, as depicted in Figure 5C. When the variation of the former (ΔEC_*integration*_) is higher than the latter (ΔEC_*discharging*_), the energy consumption shows a decreasing trend and reaches a minimum value until the two changes are equal. Hence, the lowest energy consumption can be obtained by adjusting the input intensity and synaptic resistor according to the demand in practical applications. It should be noted that when the neuron cell consumes its lowest energy consumption, the frequency is not the highest. Therefore, when the application requires a fast response to achieve optimal performance, the circuit needs to sacrifice some energy. Hence, compromise considerations between energy consumption and response speed are required when dealing with specific tasks.

As for neuron cells, the energy consumption per spike presents a similar tendency with increasing frequency under each *C*, which declines at the early stage, followed by growth, as illustrated in Figure 5D. The gray curve in Figure 5D further presents the projection of energy consumption under different *C* in the ZX plane. The energy consumption drops with decreasing the *C* at the same frequency. This can be attributed to the requirement for higher *V*_*in*_ to get the same frequency at a higher *C*, which induces the rise of base current flowing through *R*_*s*_, leading to the increase of energy consumption, as shown in Figure 5E. We can clearly see that the base current of 330 pF is higher than that of 100 pF under the same frequency. Moreover, to study the ultimate energy consumption under a smaller capacitor, we reduce the *C* to 300 fF by simulation, obtaining a minimum energy consumption of ∼0.52 pJ per spike under 300 fF, as shown in Figure 5F. This result indicates that a sub-pJ spike operation is easy to reach based on such a TS-based neuron by further reducing the parasitic *C*. It should be noted that the *V*_*th*_, *V*_*hold*_, HRS, and LRS also affect the spike energy, which is not presented in this work. The results show that the energy consumption per spike decreases with reducing the V_*th*_, V_*th*_-V_*hold*_ window, also decreases with enhancing the values of HRS or diminishing the values of LRS.

### Spiking Neural Network With Nbo_*X*_-Based Leaky Integration-and-Fire Neurons for Cart-Pole Control Through Reinforcement Learning

**FIGURE 6:**
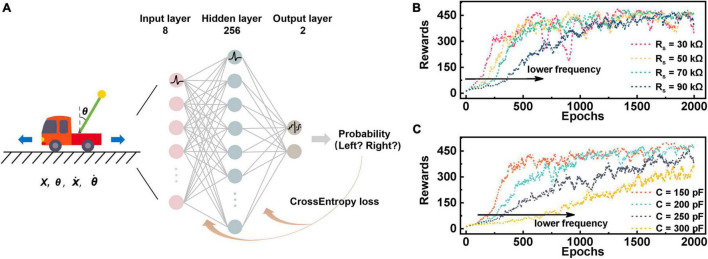
Cart-pole realized in a 3-layer SNN constructed by LIF models. **(A)** Schematic of the 3-layer SNN for cart-pole. The input of the network is four state variables. The neurons of the input layer and the hidden layer implement the established LIF model, and thereby generated pulse event sequences are transferred between different layers of the network. The neurons of the output layer adopts the leaky-integrator neuron model enabling solely integration but not emission, and the output accumulated voltage are transmitted to softmax function to produce the probability finally. **(B)** The agent’s rewards in 2000 epochs when the synaptic resistor (*R*_*s*_) changes. Spiking frequency of neurons increases with the increase of *R*_*S*_, so that relatively high rewards can be obtained in a shorter period and learning speed is thus accelerated. **(C)** The curve of rewards when the parallel capacitance (*C*) is adjusted. The direction indicated by the arrow reveals that the oscillation behavior is inhibited as *C* increases, resulting in a deceleration in rewards growth. Hence, learning speed can be facilitated by enhancing the firing response rate of neurons.

To further explore the potential of TS-based neurons in practical applications, we construct an SNN to perform a cart-pole control task by adjusting the moving direction of the cart in real time. Figure 6A depicts the conducted network with an 8 × 256 × 2 structure. The left part of Figure 6A presents the equipment of a cart-pole game (Barto et al., 1983; Wang et al., 2019), which consists of a cart and a pole attached by an un-actuated joint connected to the cart by a non-actuated joint. The cart moves along a frictionless track during working and is controlled by applying a force of +1 or −1 to it. In the beginning, the pole begins in an upright position, which needs to be prevented from toppling. The cart moves left and right to keep the pole upright and is awarded +1 bonus for each step. The episodes terminate when the tilt angle of the pole is more than 15 degrees from the vertical or the cart is more than 2.4 units from the center. To control the pole effectively, it is necessary to provide the optimal actions to maximize the total rewards in a specific state of the cart through the constructed network. The input of the network is the state variables of the cart-pole (observation). The observation that determines the state of the pole is a four-dimensional vector: (i) position of the cart on the track, represented by ***x***; (ii) angle of the pole from the vertical, represented by θ; (iii) cart velocity, represented by $\dot{\text{x}}$; (iv) rate of change of the angle, represented by $\dot{\theta }$. To achieve stable coding, we use two LIF neurons to encode the positive and negative values of a variable, respectively, which is why eight input layer neurons are needed for the four state variables. First, the four state variables of the cart-pole are fed into the input neurons, generating spike trains when the neurons’ membrane potential reaches *V*_*th*_. These spike trains serving as input are transmitted to the hidden layer neurons, inducing output spikes that are then propagated to the output layer. The output neurons are stimulated, leading to the increase or decline of the membrane potential that is further converted into probability distribution by softmax function. Consequently, a sample from this probability distribution is taken as the decision result. Different from the gradient descent algorithm in classification tasks, a gradient ascent algorithm is adopted here, since the maximum of the objective function is required, and then the synaptic weight is adjusted accordingly (Williams, 1992).

Figures 6B,C show the rewards of the backend learning agent in 2,000 game epochs under different *R*_*s*_ and *C*, respectively. The reward score gradually increases as the learning process goes on, finally reaching as high as 450. It is not hard to spot that the agent gets low scores in the early stage, since it has not learned a good policy function. Subsequently, the agent gradually trains the relationship between input and output based on the feedback of cart-pole output, indicating that the network has the ability to maintain balance for a long time. Noting that the learning speed is faster under a lower R_*s*_ or C, this is because that the neurons feature a higher spike frequency in these cases. These results provide a guide to accelerating the training process of the network through adjusting the firing frequency and thus lead us to design a neuron circuit by choosing decent parameters in practical applications.

## Discussion

In conclusion, we comprehensively investigated the spiking characteristics of a NbO_*x*_-based LIF neuron from both extrinsic and intrinsic parameters. The extrinsic parameters include input strength, synaptic weights, and parallel *C*. The higher the given stimulus, the higher the weight, and the smaller the *C*, the higher the frequency neuron fires. The intrinsic parameters include *V*_*th*_, *V*_*hold*_, *R_*high*_*, and *R*_*low*_ of the NbO_*x*_ device. Neurons capable of higher spiking frequency are equipped with lower *V*_*th*_ or higher *V*_*hold*_ and higher *R*_*high*_ or lower *R*_*low*_. Furthermore, to meet the practical application, we studied the relation between spike energy consumption and the frequency of neuron cells. The results show that a fixed cell possesses the lowest energy consumption at a fixed frequency, whatever the connected synaptic resistor is. Besides, given that the lowest energy consumption and highest frequency cannot be obtained simultaneously, it is necessary to make a trade-off between performance maximization and energy saving in practical applications. In addition, when the *C* of neuron cells decreases, the energy consumption of a single spike further decreases and could be as low as 0.52 pJ at 300 fF. Finally, to verify the feasibility of improving learning speed by optimizing device spiking frequency, we construct a three-layer SNN based on such a neuron for cart-pole control, obtaining a reward score as high as 450. These results provide important ideas and guidance for optimizing TS-based neurons and promoting them in real applications.

## Data Availability Statement

The raw data supporting the conclusions of this article will be made available by the authors, without undue reservation.

## Author Contributions

YD and XZ designed the experiments, conducted the electrical measurement, contributed to the data analysis, and prepared the article. YD and YZ conducted the simulation. PC fabricated the TS device. XZ, ZY, and QL supervised the research. All authors discussed the data and revised the text.

## Conflict of Interest

The authors declare that the research was conducted in the absence of any commercial or financial relationships that could be construed as a potential conflict of interest.

## Publisher’s Note

All claims expressed in this article are solely those of the authors and do not necessarily represent those of their affiliated organizations, or those of the publisher, the editors and the reviewers. Any product that may be evaluated in this article, or claim that may be made by its manufacturer, is not guaranteed or endorsed by the publisher.
