# Supplementary material for: Engineering Spiking Neurons Using Threshold Switching Devices for High-Efficient Neuromorphic Computing
Source: Front Neurosci. 2022 Jan 5;15:786694. doi: 10.3389/fnins.2021.786694 (PMC8766734; doi:10.3389/fnins.2021.786694)
Supplement: Supplementary file 1 [file Data_Sheet_1.docx]

Supplementary Material

Engineering spiking neurons using threshold switching devices for high-efficient neuromorphic computing

**Ding et al.**


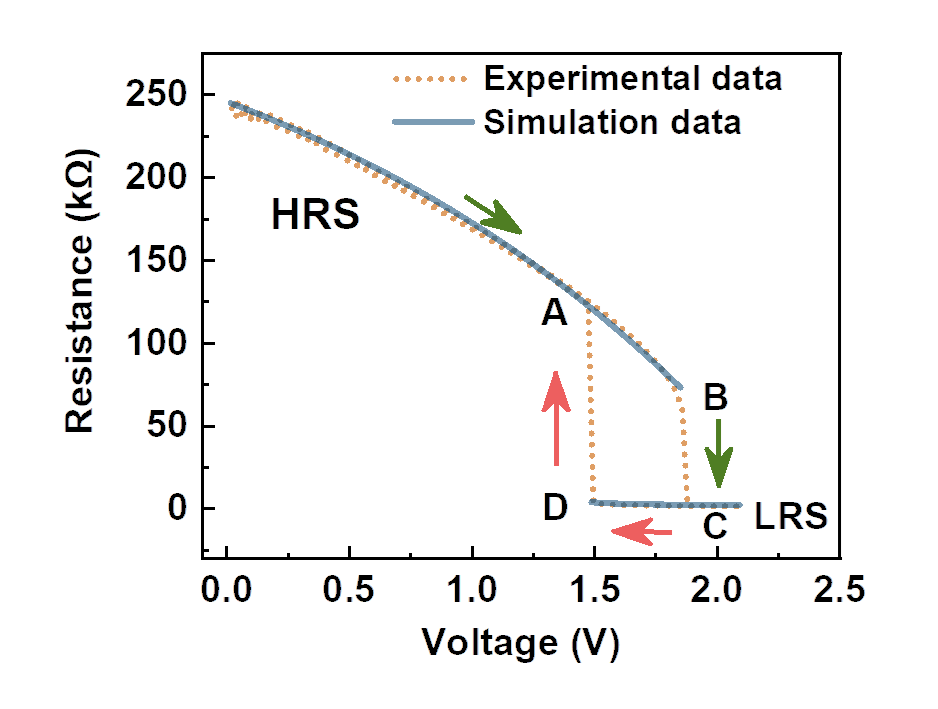


**Supplementary Figure 1.**R-V characteristics of the NbO_x_ device. The orange data points are experimental results, and the blue curves show the simulation result. When the voltage applied to the device surpasses *V_th_*, the deviceabruptlyswitches from the HRS to the LRS (from B to C). Then, the device’s resistance experiences a tiny reduction (from C to D) as the voltage across the device gradually falls off andabruptly switches back to the HRS (from D to A) once the voltage drops below *V_hold_*.


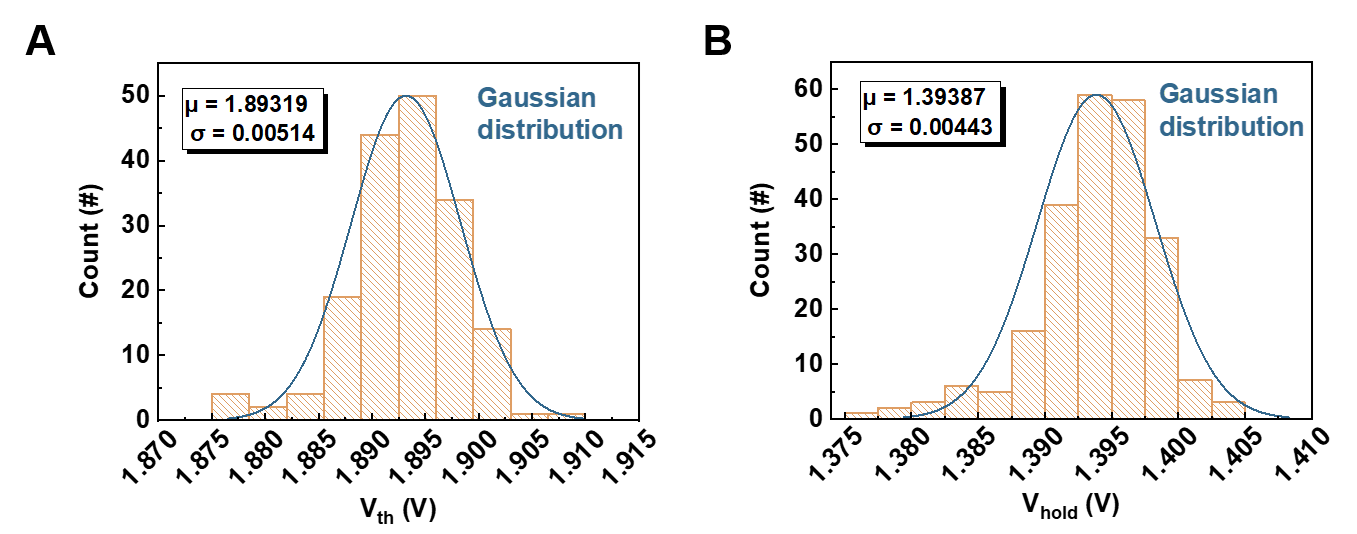


**Supplementary Figure 2.**The distribution of *V_th_*(A) and *V_hold_*(B) of the device,which conforms to obey Gaussian distribution.


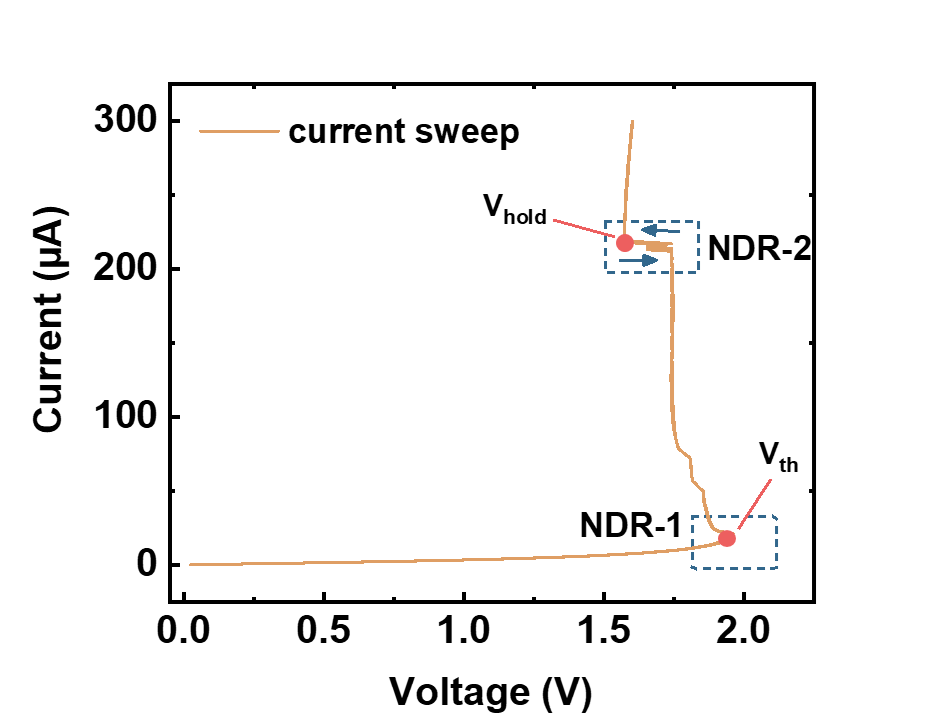


**Supplementary Figure 3.**I-V curve of the NbO_x_ device under current sweep. Two regions containing negative differential resistance (NDR) are observed. The current-controlled NDR-1 results from the instability of non-linear Poole-Frenkel (PF) transport. TheMott switching-driven hysteresis causes the temperature-controlled NDR-2. Both types of NDR are driven by Joule-heating (Kumar et al., 2013; Kumar et al., 2017a; Kumar et al., 2017b).


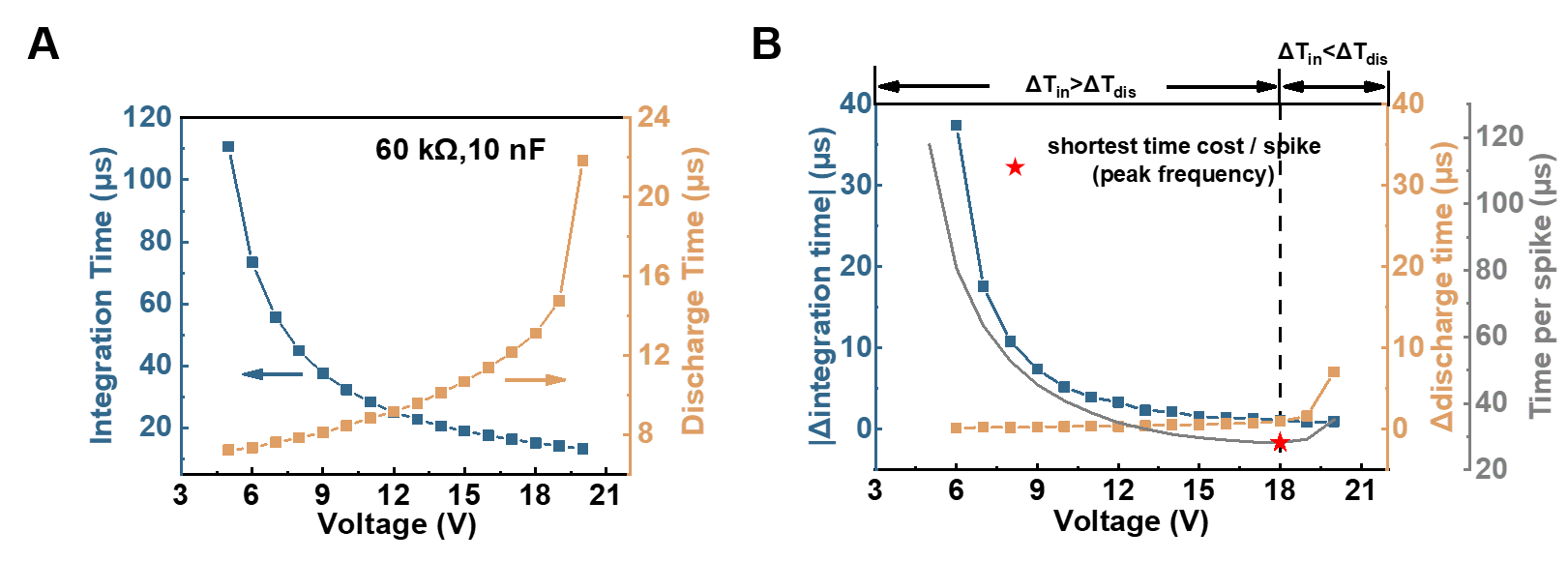


**Supplementary Figure 4.** (**A**) The variation of integration and discharge time with increasing*V_in_*. Integration timepresents an reductiontendency with the increase of *V_in_*, while discharge time continues growingup. (**B**) The variation of |*Δ integration time*| and *Δ discharge time*with *V_in_* obtained from (**C**). ‘*Δ*’represents the changed value and ‘||’ represents the absolute value. *T_in_* and *T_dis_* are abbreviations for integration time and discharge time,respectively. At the beginning stage, |*Δ T_in_*|is larger than *T_dis_*, resulting in the decrease of frequency until |*Δ T_in_*| equals to *Δ T_dis_* to spend the shortest time per spike, obtaining the peak frequency.


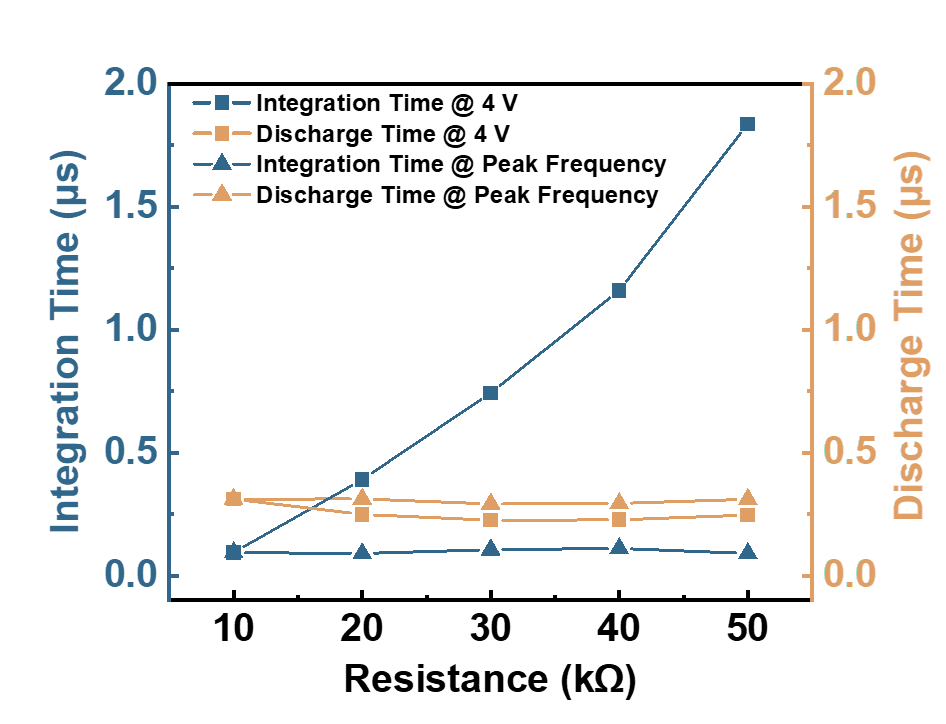


**Supplementary Figure 5.** Variation of integration time and discharging time with increasing*R_S_*under the 100 pF C.The blue and yellow lines with rectangle symbols describe the integration time and discharging time of different*R_s_* under the same input conditions, respectively, while lines with triangular symbols describe the integration time and discharging time at the peak frequency corresponding to different *R_s_*. When applying identical *V_in_*, the integration time increases with higher *R_s_* and the discharge time almost remains constant, owing to that *R_s_* is directly related to the time constant of the integration process, which indicates that increasing *R_s_* will slow down the charge build-up, hence lead to the decline of the spikefrequency.For each *R_s_*, the firing frequency increases gradually to the peak with the enhancement of *V_in_*. According to the previous discussion, the integration time decreases while the discharge time increases, corresponding to rectangle symbol plots switched to triangle symbol plots. When different *R_s_* are connected in the circuit, the integration time and discharge time at the peak frequency are almost identical, so the *R_s_*does not affect the peak frequency.


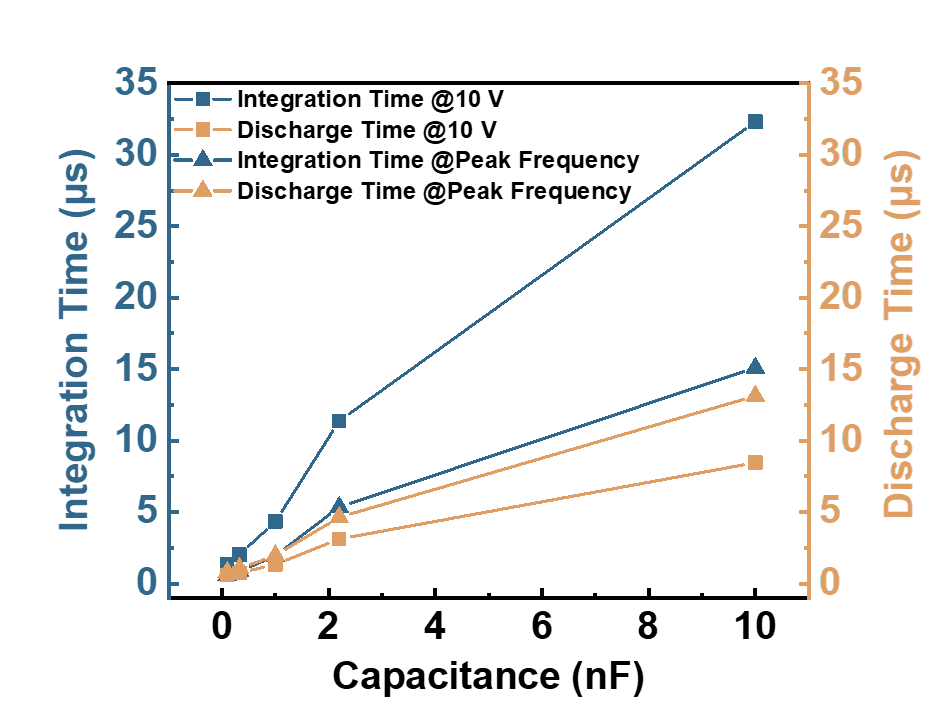


**Supplementary Figure6.**Variation of integration time and discharging time with increasing *C* under 60 kΩ R_s_. The blue and yellow square symbol curves describe the influence of C on the integration time and discharge time under the same input condition (of 10 V).The time constants of both integration and dischargeprocesses rise with the increase of capacitance, so the charging time and discharge time continue to extend, leading to the continuous decay of oscillation frequency. Blue and yellow curves embedded with triangular symbols describe the change of integration time and discharge time at peak frequency as a function of C. As *V_in_* increases, the charging loop speeds up for each C, and the discharge process attenuates gradually until meeting the balance to produce peak frequency.However, the increase of charging and discharge time constant caused by larger C cannot be offset by strengthening *V_in_*, hence the peak frequency reduces significantly.


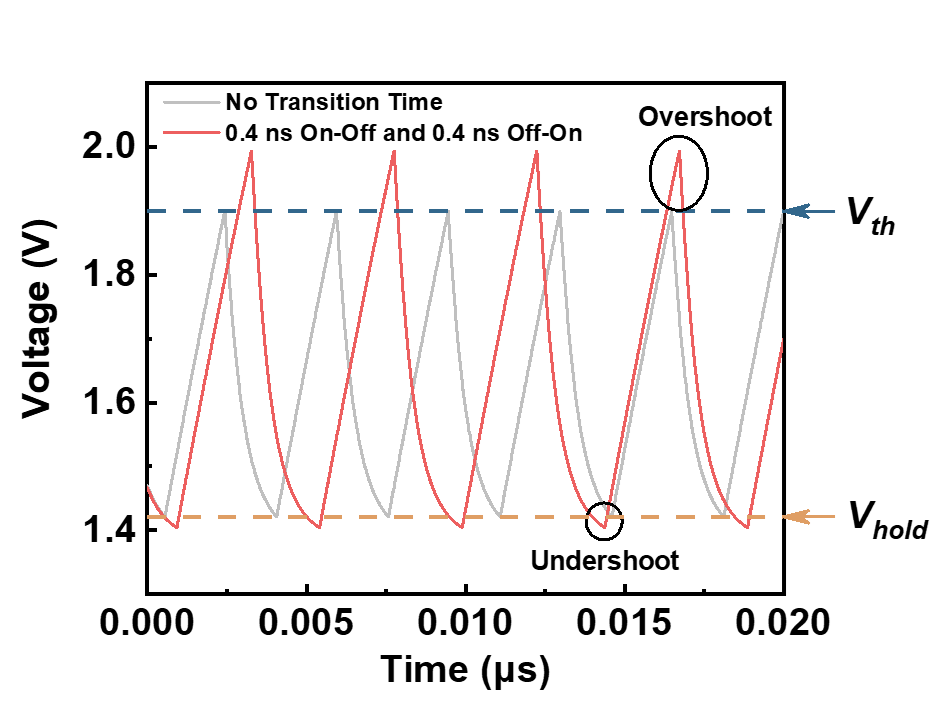


**Supplementary Figure 7.**Oscillation output of LIF model with a transition time of 0.4 ns on-off and 0.4 ns off-on of the NbO_x_ device. Circuit parameters are as followed: *R_s_* = 60 kΩ, C = 1 pF.An overshoot of the voltage charging above the *V_th_* and an undershoot of the voltage discharging below the *V_hold_*are observed. This is because that the transition time of the device is comparable to the integration (discharge) time.


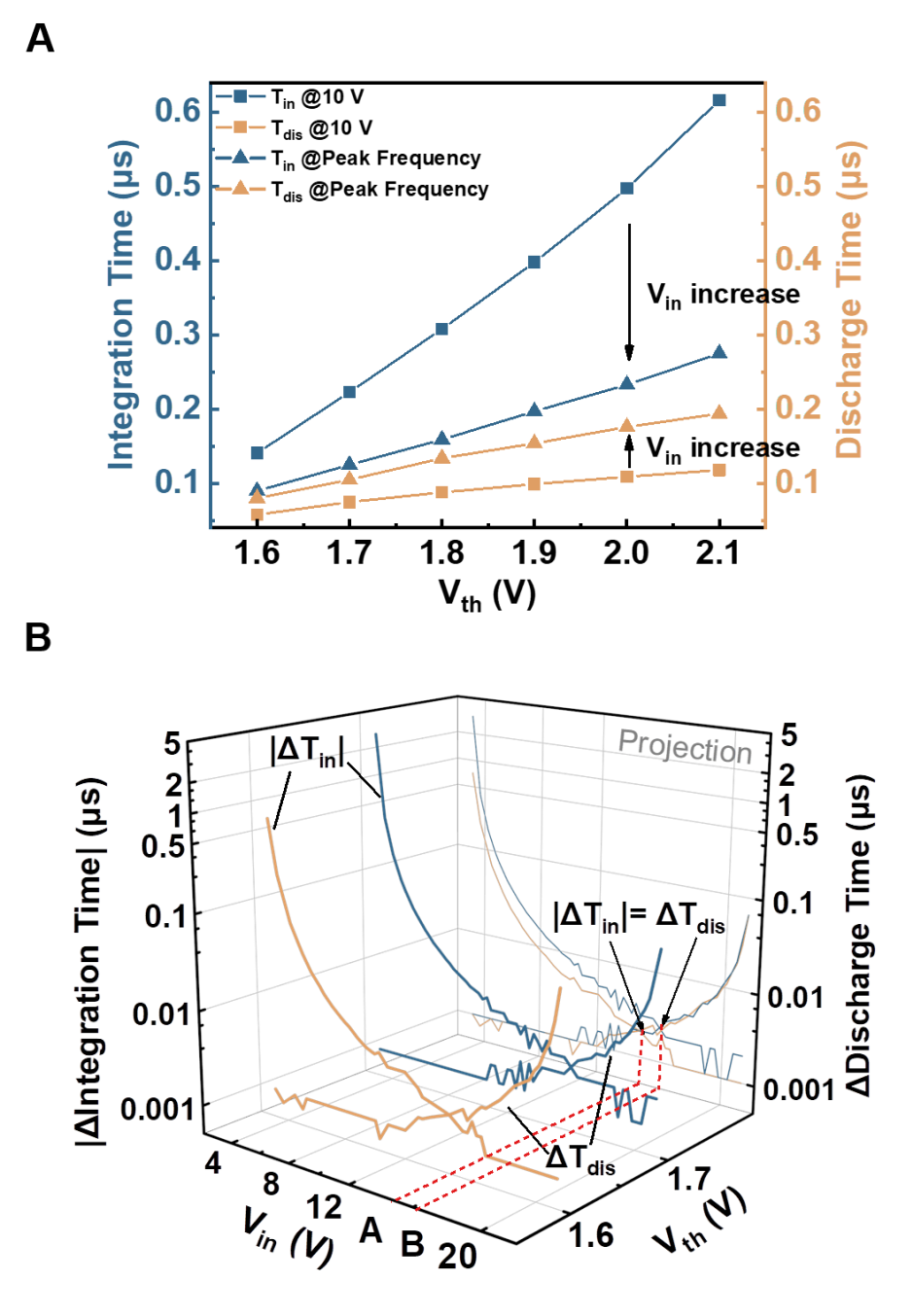


**Supplementary Figure 8.** (**A**) Variation of integration time and discharging time with increasing*V_th_*. The blue and yellow symbol curves describe the integration time and discharge time varied with the *V_th_* of the device under the same input condition (of 10 V). The increase of *V_th_* prolongs both the charging process of C from *V_hold_* to *V_th_* and the discharge process from *V_th_* to *V_hold_*, thereby the frequency tends to falloff. Blue and yellow plots with triangular symbols depict the integration time and discharge time at respective peak frequencies corresponding to each given *V_th_*, both increase with enhanced *V_th_*, i.e. higher *V_th_* corresponds to lower peak frequency. (**B**) The change of integration time and discharge time as a function of *V_th_*.The yellow and blue curves demonstrate the relationship between the variation of integration and discharge process in a single spike with *V_in_* when *V_th_* is 1.6 V and 1.7 V, respectively. Under each *V_th_*, the peak frequency is achieved at the intersection point when the decrease of integrationtime equals to the increase of discharge time. As can be seen from the mapping curve on the ZX plane in the figure, a higher *V_th_* result in a right shift of the abscissa of intersection, i.e. a larger *V_in_* is required for saturation point.


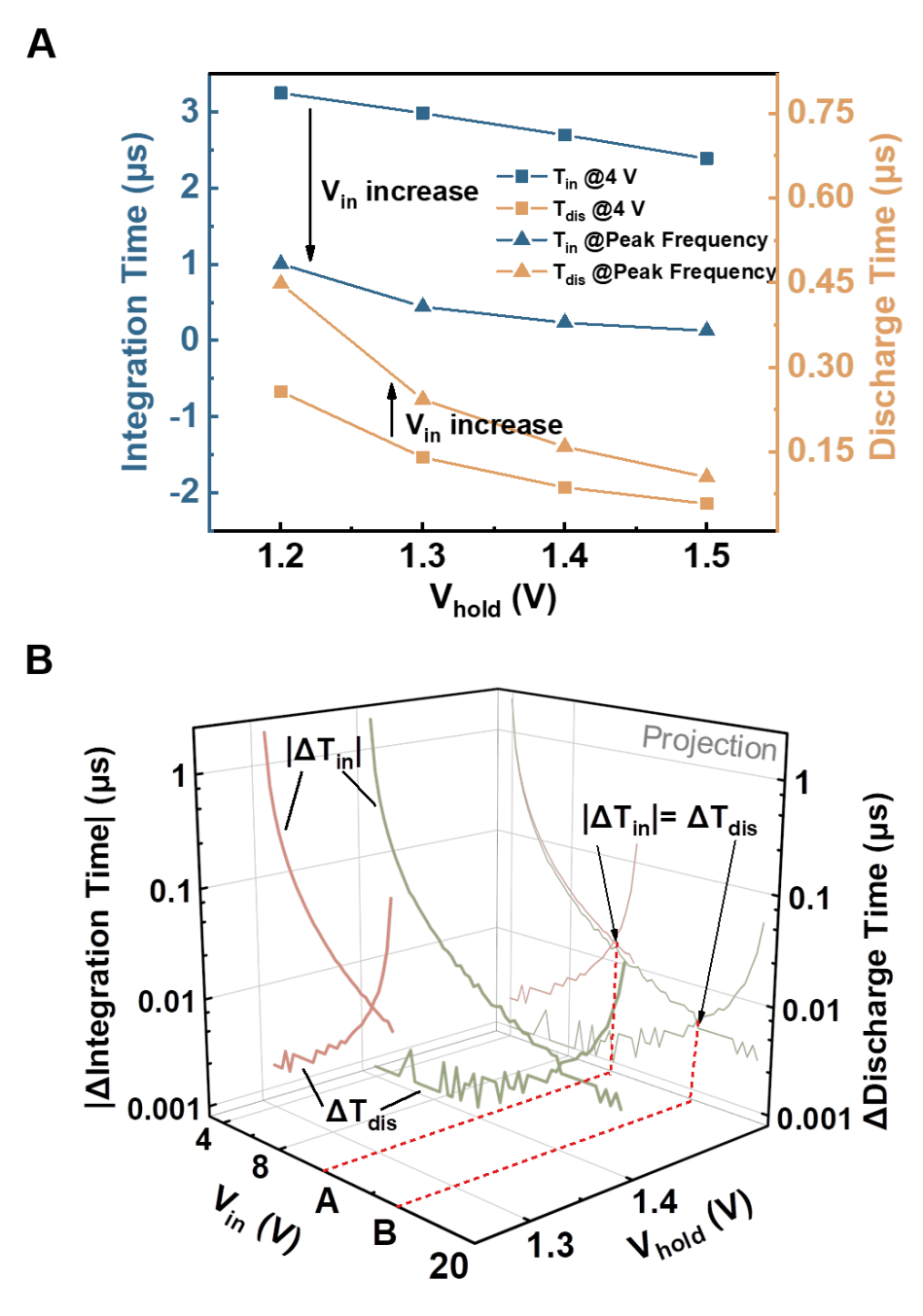


**Supplementary Figure 9.** (**A**)Variation of integration time and discharging time with varying*V_hold_*. Symbol plots with rectangles indicate that the two stages of a single spike, the transitions from *V_th_* to *V_hold_* and from *V_hold_*to *V_th_* are both accelerated with higher *V_hold_*, leading to a rise of frequency under the same *V_in_*.When *V_in_* increases to generate the saturation, the integration and discharge time decay with the increase of *V_hold_*, consequently leading to an increase of peak frequency. (**B**) The change of integration time and discharge time as a function of *V_hold_*. The decline of both |Δintegration time| and Δdischarge time brought about by increasing *V_hold_* (from pink curves to green curves) postpones the arrival of frequency saturation point, requiring the device to generate a peak frequency at a higher *V_in_* (from A to B).


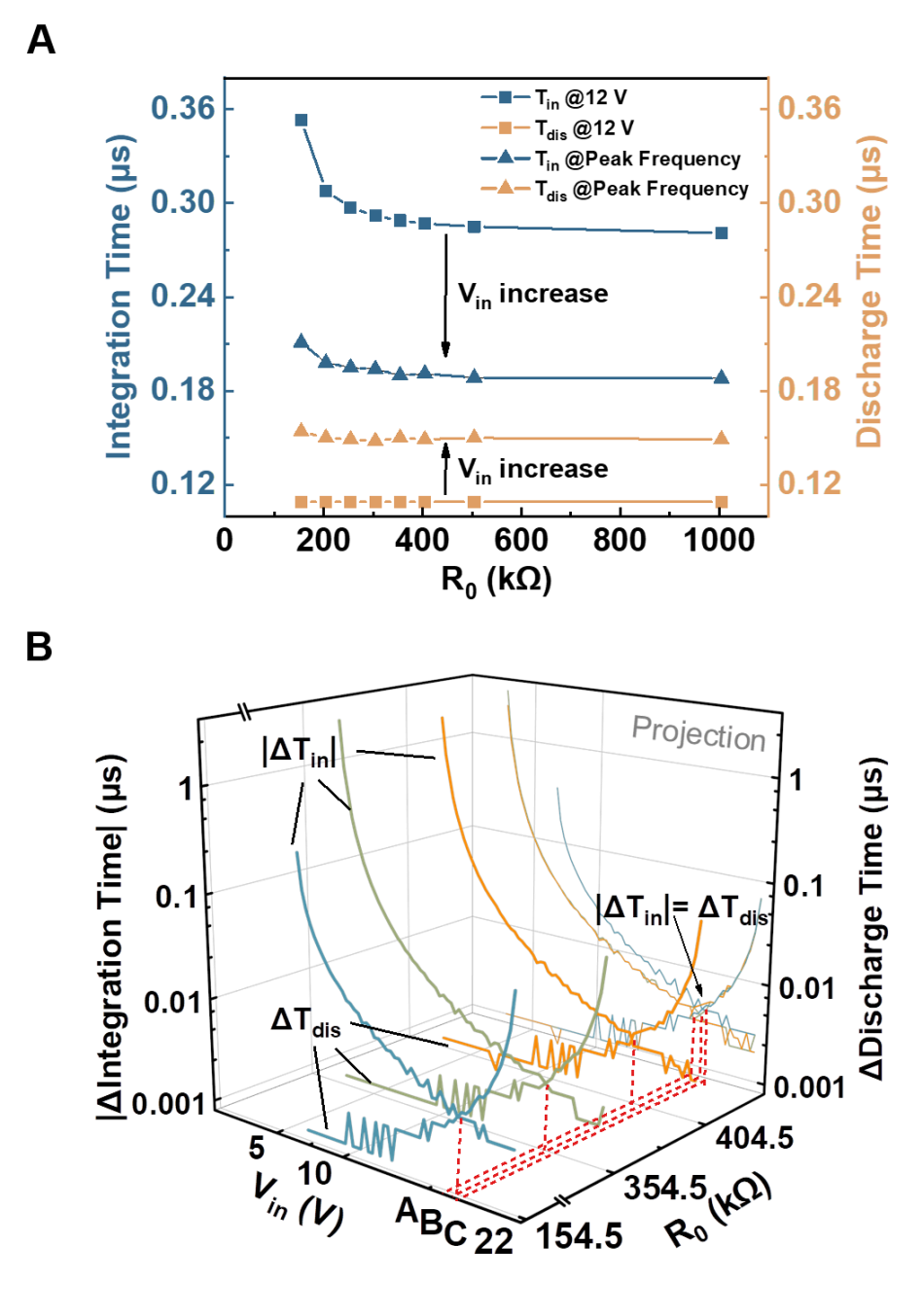


**Supplementary Figure 10.** (**A**)Variation of integration time and discharging time with increasing *R_0_*. A larger *R_0_* reduces the partial voltage across *R_s_* and thus reduces thecurrent through *R_s_*, hence slowing down the charging process and further preventing the firing behavior within identical *V_in_*. Actually, the shrinking effect of integration time by *R_0_*is equivalent to which by *R_s_* in principle. When reaching the respective peak frequency for each given *R_0_* through heightening *V_in_*, the integration time first declines followed by approaching saturation, while the discharge time is nearly unchangedwith the increase of *R_0_*. This indicates that the peak frequency is almost unrelated to *R_high_*, especially when *R_0_* is raised to a very high level. (**B**) The change of integration time and discharge time as a function of *R_0_*.The x-coordinate of the intersection of three lines (blue, green, and yellow) respectively corresponds to points A, B, and C, demonstrating that the *V_in_* required for generating peak frequency tends to increase slightly.


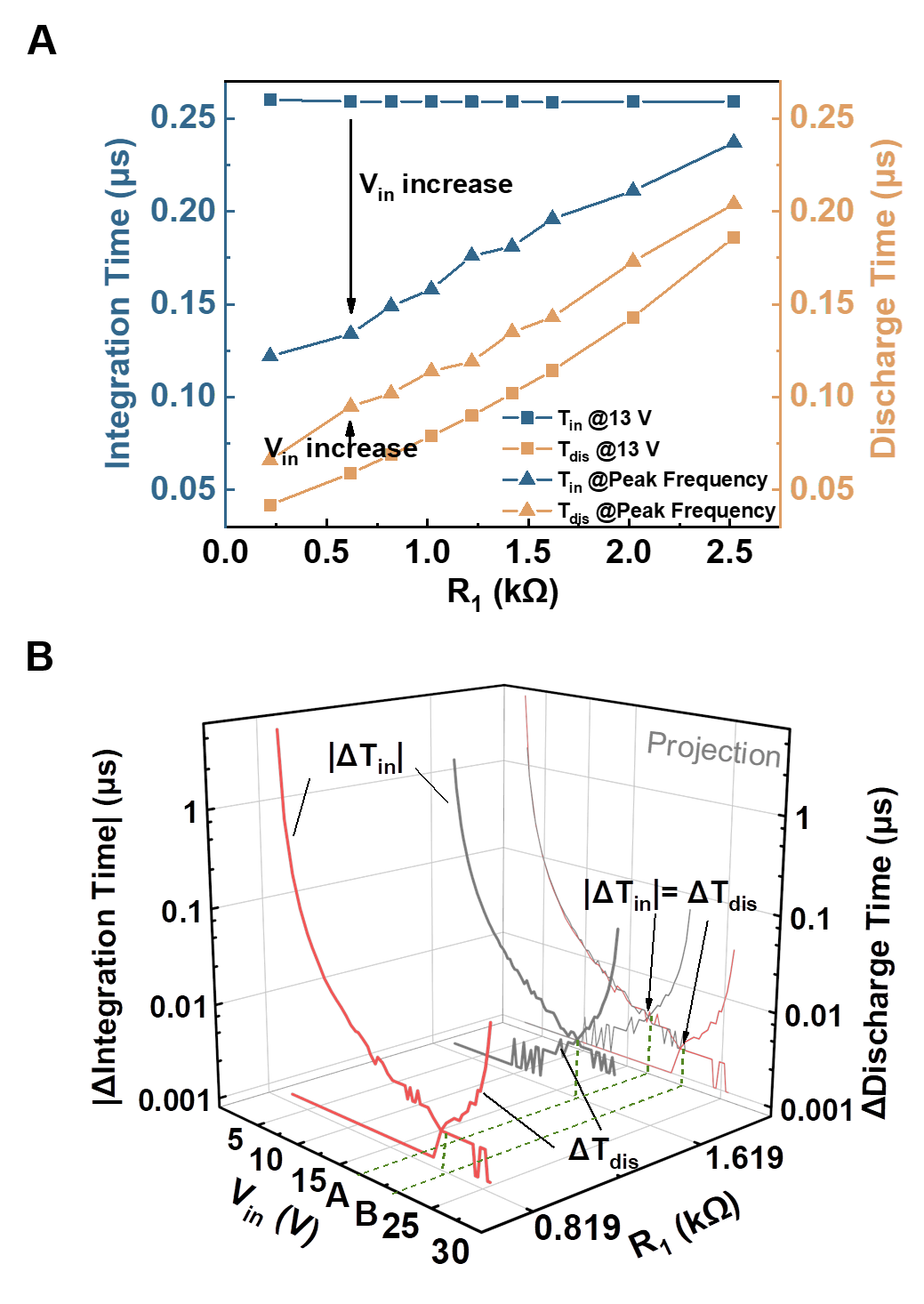


**Supplementary Figure 11.** (**A**)Variation of integration time and discharging time with increasing*R_1_*. Unlike R_high_, which mainly affects the charging loop’s time constant, R_low_ makes a difference to the time constant of the discharge process under the same *V_in_*, while the integration time is impervious to *R_low_*. With the increase of R_1_, the discharge current gradually decreases, prolonging the discharge process. For each *R_1_*, the frequency gradually increases to saturation as *V_in_*intensifies. Both the integration and discharge time corresponding to the peak frequency increase with the growth of *R_1_*. Thus *R_1_* affectsthe peak frequency.(**B**) The change of integration time and discharge time as a function of *R_1_*. The projectionon the ZX plane indicates that the extension of the discharge time itself caused by a higher *R_1_* at the same V_in_ further leads to the increase of Δdischarge time, thereby hastening the arrival of the crossover point and reducing the voltage required to obtain the peak frequency (from B to A).

# References

Kumar, S., Pickett, M.D., Strachan, J.P., Gibson, G., Nishi, Y., Williams, R.S. (2013). Local temperature redistribution and structural transition during joule-heating-driven conductance switching in VO2. *Adv Mater*. 25(42), 6128-6132. doi:10.1002/adma.201302046.

Kumar, S., Strachan, J.P., Williams, R.S. (2017a). Chaotic dynamics in nanoscale NbO 2 Mott memristors for analogue computing. *Nature*. 548(7667), 318-321. doi:10.1038/nature23307.

Kumar, S., Wang, Z., Davila, N., Kumari, N., Norris, K.J., Huang, X., et al. (2017b). Physical origins of current and temperature controlled negative differential resistances in NbO2. *Nature communications*. 8(1), 1-6. doi:10.1038/s41467-017-00773-4.
